# Unmet health‐care needs and human rights—A qualitative analysis of patients' complaints in light of the right to health and health care

**DOI:** 10.1111/hex.13038

**Published:** 2020-02-18

**Authors:** Annelie J. Sundler, Laura Darcy, Anna Råberus, Inger K. Holmström

**Affiliations:** ^1^ Faculty of Caring Science, Work Life and Social Welfare University of Borås Borås Sweden; ^2^ School of Health, Care and Social Welfare Mälardalen University Västerås Sweden; ^3^ Department of Public Health and Caring Sciences Uppsala University Uppsala Sweden

**Keywords:** health services, human rights, patient preference, patients, qualitative research, right to health

## Abstract

**Background:**

This study focuses on patient complaints from a human rights perspective. Despite the UN Convention on Human Rights being widely recognized, it has not previously been examined in relation to patients’ complaints on health care. A human rights perspective and the right to the highest attainable standard of health are a major sustainability challenge in health care today. Previous research points to patients’ complaints as a growing concern for health‐care organizations, and the handling of this concern can lead to improvement in health‐care services.

**Objective:**

The aim was to analyse patients’ complaints on health‐care services and to examine expressed needs for health care from a human rights perspective.

**Methods:**

In this descriptive study, a random sample of 170 patient complaints about Swedish health‐care services were qualitatively analysed from a human rights perspective.

**Results:**

The complaints are described in three themes: the right to available and accessible health‐care services, the right to good quality health‐care services and the right to dignity and equality in health care. Questions of availability, accessibility, acceptability and quality are highlighted by patients and/or relatives making complaints on health‐care services.

**Discussion and Conclusion:**

This study emphasizes the human right to health in relation to patient complaints. Findings indicate that this right has been breached in relation to availability, accessibility, acceptability and quality in health‐care services. Further debate, education and investigations are necessary to ensure that patients’ rights to health and health care not be taken for granted.

## 1 BACKGROUND

There are few things in life as important as health. The right to health requires that health‐care service guidelines, goods and facilities are available, accessible, acceptable and of good quality.^1^ The importance of health‐care services to quality of life is well recognized in modern society. Health is a fundamental part of the UN Conventions of Human Rights and indispensable for the exercise of other human rights. People will get ill, accidents arise and health‐care facilities be required to diagnose, manage and treat various diseases. Despite the great importance of health‐care services and substantial efforts made to safeguard the quality of health care, there are patients suffering from medical and patient safety errors.^2^, ^3^ The significance of preventing harm to patients and minimizing incidents and errors has been underscored over the last few decades.^4^

Patient complaints and understanding patients’ experiences have been addressed universally as a growing concern for health‐care organizations. Such complaints can indicate problems within health‐care services and considered important for feedback.^5^ Patient complaints can help improve health‐care services,^6^ as drawing attention to previous mistakes may help preventing such mistakes or incidents from being repeated.^7^ However, it can be stressed that formal patient complaints are relatively rare.^7^ A study on cancer patients that experienced a preventable, harmful event reported that the patients seldom formally reported such experiences.^8^ Moreover, a study on patient complaints in the Dutch regulatory system indicated that only few of those making complaints felt that it had led to improvements.^9^ Gallagher and Mazor^7^ mean that the patient's perspective has been discounted in favour of the medical perspective of health‐care providers. They argue that the perspective of patients and family members can in fact provide important insights into health care, particularly when health care is complex, fragmented or rife with transitions. Thus, patients and their families can help detect lapses in safety or quality of care. In spite of this knowledge, there is a dearth of studies focusing on problems reported directly by patients. Furthermore, knowledge on understanding health care in clinical practice in relation to a human rights perspective is still limited.

Today, there is a strong emphasis on patient participation and patient/person‐centred care.^10^ Although person‐centred care has been widely advocated, challenges remain on how to translate person‐centred care into clinical practice.^11^ The needs and rights of patients need to be acknowledged. A recent study points to the prevalence of a dominant paternalistic discourse in Norwegian legislations on health care and raises the issue of the power of medical and health‐care systems over the needs and rights of patients.^12^ In Sweden, where this study was conducted, the Health and Medical Service Act^13^ and the Patient Act^14^ guide health‐care services. Swedish health care is by law^13^ stipulated to provide good and equal care for all citizens. However, the number of health‐care providers differs considerably between urban and rural areas. In northern Sweden, for instance, there are few health‐care providers and patients might have to drive several hours to visit a doctor. Hence, access is not equal for all. Swedish health care is primarily publicly funded and managed by tax revenues collected by the county councils. However, privately managed health‐care facilities exist, especially in larger cities, which are also publicly funded. Like many other health‐care systems, Swedish health care has been strongly influenced by the New Public Management reform trend which is associated with objectives of efficiency, cost control and performance evaluation. Currently, Swedish health care is struggling with issues such as accessibility, quality, finance and shortages of staff. Patient safety is a prioritized issue, as seen with the Patient Safety Act,^15^ which gives patients and family members opportunities to influence the quality of health care. Additionally, the Patient Act^14^ aims to strengthen patients’ integrity, participation in care and self‐determination. Hence, patients in Sweden have rights. However, the Swedish legal system does not allow patients to claim these rights from health‐care providers.

The right to health has been debated in relation to global health, in particular on issues surrounding universal coverage vs universal access.^16^ The concept of universal coverage is a utopian idea that does not necessarily translate into a right or access to health. The right to health is elaborated on in Article 12 of the International Covenant on Economic, Social and Cultural Rights that states the ‘right of everyone to the enjoyment of the highest attainable standard of physical and mental health’.^17^ The highest attainable health has been discussed as a practical possibility, and whether or not there can be a right to health.^18^ Brudney^18^ elaborates that ‘health care is only one important need amongst others, and in a world of limited resources, not everyone can have all things good’ (p. 249). By associating health with financial constraints, the notion of health as a biological right, as it was first defined by the WHO,^16^ is undermined. The right to health is a complex challenge, and governments have an obligation to progressively realize this right. Social rights in the Netherlands are internationally well‐known but like in Sweden, not considered enforceable. No human being is ever under the obligation to receive or accept care, but governments need to ensure equal access to health care for all individuals. In countries such as the Netherlands, it is increasingly the case that health care depends on factors other than medical conditions. The dominant principle of health‐care law in the Netherlands is one of the personal autonomy. In fact, Dutch law sees individual self‐determination as a right to which every human being is entitled.^19^ Questions remain on how to understand and move towards the full realization of the right to health and health care. It may not be possible to specify this right in any reasonable and clear way.

In this study, we apply a human rights perspective, including the right to the highest attainable standard of health, when examining patient complaints in Swedish health care. A previous study emphasizes patients’ exposure and vulnerability in relation to complaints and problems experienced from Swedish health‐care services.^6^ In the present study, patient complaints of situations and experiences from health care are elaborated on retrospectively from a human rights perspective in relation to patients’ conditions and treatments. What can we learned from patients making complaints on health care from a human rights perspective?

### 1.1 Aim

The aim of this study was to analyse patients’ complaints on Swedish health‐care services and to examine expressed needs for health care from a human rights perspective.

## 2 METHOD

### 2.1 Research design

A descriptive design with a qualitative approach was used. The study was conducted having obtaining permissions and approvals for the research, as part of a more comprehensive study on patient complaints and human rights. Patient complaints were analysed with a method for thematic analysis,^20^ in relation to human rights and the right to health. A previously published paper has reported on the content of the complaints.^6^

### 2.2 Setting and sample

In this study, a sample of patient complaints collected from a patients’ advisory committee (PAC) in Region Västra Götaland, Sweden, was analysed. Swedish PACs assist patients and/or relatives with complaints related to publicly financed health‐care services both in hospitals and outside hospitals. The health‐care services in Region Västra Götaland provide health care and medical treatment for over 1.7 million people. The Region operates 17 hospitals, 121 health‐care centres and 170 public dental care centres. Subcontracted private centres provide some of the care.

In 2016, a systematic random sample was taken of 170 complaints made by patients and/or their relatives from 5689 patient complaints registered in the system in 2015. Researchers accessed data with the help of a legislative controller at the actual PAC. All patient complaints were anonymous to the researchers.

The inclusion criteria were patient complaints made in 2015, including a narrative written by a patient or a relative and sent by mail or e‐mail to the PAC. This leads to a sample of 938 complaints. From these, a systematic random sample was made with regard to complaints received on the 5th, 10th, 15th, 20th and 25th of every month. This resulted in a sample of 170 patient complaints used for the in‐depth qualitative analysis.

**Figure 1:**
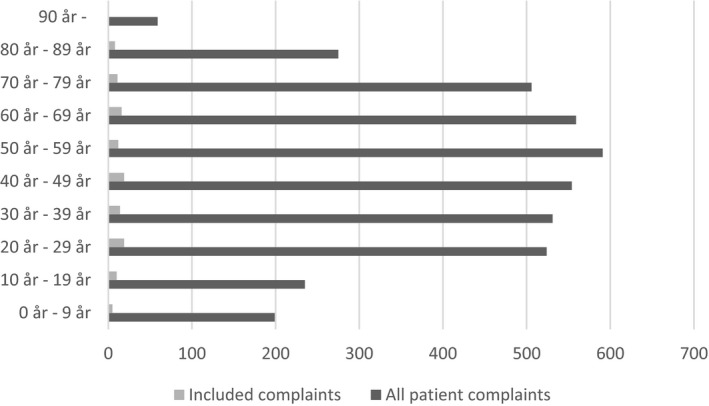
Age of the patients in the complaints (n = 5689). Age was unknown in 1656 of all complaints and in 55 of the included complaints

The complaints related to hospital care (61%), primary health care centres (24%), dental care (6%) and other health‐care services (9%). The patients were predominantly female (53%). For the distribution of age, see Figure 1. The proportion of the above variables in the sample were comparable to the proportion of similar variables in all complaints (n = 5689), respectively.

### 2.3 Data analysis

A method for thematic analysis was used as described by Sundler et al^20^ where themes were derived from experiences reported by patients and/or relatives making complaints on health‐care services. A human rights perspective guided the analysis to interpret meanings from the data. The following four steps describe the analysis process.

Step 1: Firstly, to establish familiarity with the data, the researchers open‐mindedly read all data several times.
Step 2: The reading continued with a systematic search for meaning units in the data on experiences and needs described in the complaints by patients and patients’ relatives. While re‐reading all text, the researchers reflected on details in the text to allow new insights to emerge. To identify and sort meaning units, parts of the text representing meanings were marked. The analysis proceeded with comparisons of these meanings for emerging patterns of differences and similarities.
Step 3: Meanings were condensed in a descriptive text. During this process, descriptions were developed and labelled in themes. The themes and their meanings were further explored and interpreted in view of the right to health and a human rights perspective, for example with the goal of attainment of highest possible standard of health where health‐care service guidelines, goods and facilities are available, accessible, acceptable and of good quality.
Step 4: Finally, the analysis was organized into a meaningful wholeness with three themes describing the meanings of experiences from health care in patients complains in relation to the right to health. Examples from the original data were used in the results to illustrate that themes and interpretations were grounded in the data.

The analysis was mainly performed by the first author. During the process, the analysis was further refined and discussed among all researchers. We reviewed and discussed how to understand meanings in the complaints and how these meanings could be interpreted in relation to the right to health.

## 3 RESULTS

Three themes describe the analyses of experiences of health‐care services derived from patient complaints and elaborated on with a human rights approach:

- The right to available and accessible health‐care services.
- The right to health‐care services of good quality.
- The right to dignity and equality in health care.

Each of the themes is further described below and illustrated with some examples from the complaints made.

### 3.1 The right to available and accessible health‐care services

Needs for health care may be of uppermost importance following illness and/or a diseases. In an optimal situation, the health and medical care offered corresponds to the needs and expectations of the citizens. However, experiences were described regarding problems of accessing health care when needed. Such experiences were related to unmet needs in both short‐term and emergency situations, as well as in long‐term care and treatment. The examples given here illustrate situations where different unmet needs were evident:

- An elderly couple call for an ambulance following the man having fallen and then fainted at their apartment. The man hit the back of his head as he is falling and does not remember anything from the incident. When the ambulance arrives, health‐care personnel decide not to allow the man to be transported by the ambulance to the emergency department. Neither is the physician contacted by phone. The man is left in their home, as the ambulance staff considered him to be intoxicated by alcohol. However, he is not. Rather, his condition was caused by a cerebral haemorrhage observed several days later when another ambulance is called for. The second call to emergency services follows a second fall and fainting attack after which the elderly man begins to cramp. His condition has worsened and he requires intensive care as a result of the cerebral haemorrhage he most likely received when falling the first time. (Complaint 83)
- An adult relative reports repeated cancellations on an elderly parent for planned surgery at repeated hospital visits. At two different hospital admissions, the elderly parent has undergone preoperative preparation, including fasting, and then has to go home on the day for the planned surgery without any operation due to staffing issues. Since the diagnosis of hydrocephalus six months ago, the elderly person's general condition has deteriorated pending the operation. The adult relative is upset, disappointed and angry. They feel their elderly parent is repeatedly exposed to unnecessary suffering, while the original health condition deteriorates. In the complaint, they ask which rights and legal requirements their parent has. (Complaint 98)
- A patient who previously moved from one county council area to another has made a complaint regarding significant short comings in health‐care services for psychiatric outpatients. The patient states getting neither access to a doctor's appointment or medical treatment and care for several months. As time goes by, the patient describes it as impossible to get in contact with a psychiatrist, despite several attempts to address the patient's needs. The patient has never previously experienced such problems and is troubled and upset and feels a complete lack of control with the situation. Lack of contact with psychiatric treatment services means that the patient is lacking the necessarily medical certificate for sick leave and is now is without an income. Responses from health‐care services allude to staff shortages. (Complaint 38)
- A parent has made a complaint regarding follow‐up treatment of their child's congenital clubfoot. The child's clubfoot was continuously followed up by a specialist for the first five years. Thereafter, no follow‐up has taken place. Now, four years later, the child has grown and problems arisen so the parents have contacted specialist care services. They are informed that there is a waiting time of at least seven months to meet a specialist. The parents ask whether or not there should have been regular follow‐ups to avoid the problems now arising and the consequently suffering with pain and difficulties in walking the child has experienced from these problems. (Complaint 17)

The complaints made point to complex challenges related the right to health and available health‐care services. Health is an important resource for society and for people to be able to live a good life. State bodies have an obligation to incorporate the Convention on the Right to Health in standard practices and to create the best possible conditions for health and health care for everyone. This includes functioning health‐care facilities and the availability of goods and services in sufficient quantity.^17^ The complaints made seem to be related in particular to the availability of health‐care services. Although Sweden, as with many welfare states, is quite developed in relation to levels of health and health care as described in the Convention, questions remain regarding what levels are acceptable and what remains to ensure the Convention is fully put in place.

Health‐care services need to balance between adhering to the needs of users and available resources. Problems related to acute care, the provision of continued care outside hospitals for persons with on‐going health‐care needs and in particular for persons with mental health problems were observed in the data. The situations above illustrate an imbalance between individual needs and available health‐care services, where patients suffered and their health was negatively affected. In some complaints, already limited health‐care services were used additional times, leading to deteriorated health conditions, additional use of health‐care services and increased costs for society. Interestingly, shortcomings in health‐care services do not solely result in suffering for individual patients, but can also be cost inefficient for the society.

### 3.2 The right to health‐care services of good quality

The right to health care also relates to appropriate and good quality health and medical care and treatment. Complaints made describe situations where patients have suffered or been injured as a direct result of health care and where there has been lack of quality or safety in the health care provided. The examples given here illustrate situations where lack of quality or safety in health care led to suffering or harm to patients:

- A next of kin describes complaints regarding an elderly person who died during a hospital admission. The elderly person was treated with warfarin, a drug that is used as an anticoagulant (blood thinner), which contributed to the patient's death from injuries sustained during a fall when admitted. The medical treatment poses a risk of bleeding which needed to be considered in all care and treatment. The patient would have required frequent observations and was of high risk of falls. The patient's death could probably have been prevented if a risk assessment of the patient's physical and mental health had been performed prior to the fall. (Complaint 131)
- Another patient reports an event with a post‐surgical wound infection which caused pain and suffering. Shortly after surgery, the patient experienced increased pain on a newly operated wrist. The patient was concerned and called post‐op services. The patient describes being rejected and referred to a visit planned several days later at the local health‐care centre. At that visit, the patient's wound is infected, several surgical stitches have burst and the wound is open. The patient was transferred to the emergency department for further treatment. Unnecessary suffering and pain could have been avoided if it had been followed up immediately. (Complaint 31)
- A patient with an aggressive lung cell cancer has made a formal complaint when not getting any further health care after radiation therapy. The patient is weak, and she is concerned for her health. For weeks, she has been waiting for a follow‐up visit to the clinic. She has tried to call the clinic for several times, but does not get any help. She is worried about test results and plans for care and treatment. Now she wants to get help in sorting out the situation. (Complaint 37)

The patient complaints describe problems regarding quality of care and patient safety. There were experiences of patients suffering the consequences of health‐care mistakes and incidents. An essential element of the right to health is quality,^17^ alluding to the fact that health facilities, goods and services must be scientifically and medically appropriate and of good quality. Thus, it is important to prevent unnecessary risks and patient harm, as far as possible. When such incidents occur, it is important to learn from these situations.

Complications and breakdowns in transition of care from one context to another were found to be among the reasons for some of the patient complaints. Other complaints were related to lack of continuity in care with resulting negative impacts on patient safety and trust. Today's increasingly specialized health care, with different specialists for different health problems, can make health‐care service complex and fragmented which can lead to vagueness regarding responsibilities and obligations and problems in care transitions. According to the European Commission's Patient safety and quality of care working group,^21^ a culture that values and strives for patient safety and quality improvement should be prioritized. Important insights can be gained from patient complaints describing breaches in safety and quality of care. Quality improvement systems and risk and event analyses need to be supplemented with the views and complaints of patients themselves.

### 3.3 The right to dignity and equality in health care

The very essence of the Convention of Human Rights is respect for human dignity and freedom. It includes the right to equality and non‐discrimination. The importance of the principle is widely recognized, although there may be some confusion regarding the demands it makes on health‐care services from a patient perspective. Attitudes and approaches, including verbal and non‐verbal communication, were found to be important components of experiences related to patient dignity and equality. Patient complaints reported on problems regarding communication and interaction with health‐care professionals with descriptions of experiences of feeling devalued, dismissed and dehumanized by health‐care professionals with whom they came into contact with. The patients' vulnerability becomes clear when freedom and dignity is compromised. Below are some examples from such complaints.

- Complaints were made on the attitudes and communication of a physician. The patient experiences the physician as unstructured and non‐chalant and feels helpless and frustrated when the physician does not listen. For instance, the physician is not prepared during appointments or does not listen to the patient's descriptions of symptoms or experiences. The physician neither calls the patient when having made a scheduled telephone appointment nor completes the medical certificates needed for sick leave. When the patient contacts the head of the primary care centre where the physician works to complain, the physician has an even more ‘irritated tone’ with the patient at subsequent visits. The patient is disappointed and feels disadvantaged having complained. (Complaint 47)
- A patient complains regarding lack of confidentiality. After a fall incident, the insurance company requests access to the patients’ medical records. When the primary health care centre send the medical records to the insurance company, something goes wrong; instead of the current necessary documentation from the fall incident, the company received a copy of documentation of several years of medical visits and confident health information not relevant for the insurance company. The patient feels very offended when they discover that private information from the medical record has been disclosed. (Complaint 46)

The acceptability and quality of health‐care services include treating patients with dignity, creating trust and promoting harm. The behaviours and attitudes of professionals are a relevant aspect in this. Human rights and Swedish legislation are designed to protect dignity and humanity. Patients should be well informed and consent to care and treatment offered to them. The examples above illustrate experiences from situations where patient value and dignity were not always noticed or respected. Health‐care professionals have an obligation to safeguard dignity and equality. Patients are generally in a vulnerable position—how this is encountered and managed by health‐care professionals may either increase or decrease such vulnerability.

## 4 DISCUSSION

The findings of this study point to challenges in relation to the right to health and questions of availability, accessibility, acceptability and quality. This may be one of the few studies looking at individual patient's situations in light of the right to health. It is vital that health‐care services be debated to fulfil the right to health. A human rights perspective and the right to the highest attainable standard of health are a major sustainability challenge in health care today. Although the number of complaints was few in comparison with all available health‐care services in the Region Västra Götaland, the results point to an interesting gap between universal human rights and the circumstances of individual patients.

Human rights, as stated in the Convention, may be vague in relation to individual situations. In Article 12 of the International Covenant on Economic, Social and Cultural Rights,^17^ the right to health is elaborated on as what it could or should be. Subsequently, there is no list of issues or standards in relation to the right; rather, the right to health is elaborated upon with a States' obligations with respect to this right. The findings indicate that lack of clarity seems to undermine the patients’ rights. Hence, there is a need for future improvements, particularly regarding quality and safety of health care and a need for a more person‐centred approach towards patients. The right to health can be regarded as paramount to health‐care quality. The shortcomings in health care presented here were found in all parts of Donabedian's^22^ model for assessing quality in health care, that is in the structure of health care, in the process of health‐care provision and in the outcomes of health care. Sweden does not fully comply with the demands on human rights that have been examined here. While human rights refer the rights of individual citizens, Swedish health‐care legislation is regulated so that health and medical care is regarded as an obligation for society as a whole, not possible for patients to legally enforce. The policies surrounding health‐care service delivery and associated costs are a large part of the national discourse in many other welfare states, and in addition to Sweden, the WHO has begun to leave behind the idea of health as a human right for all as faced of huge global health inequalities and rather discusses universal coverage vs universal access.^16^ This raises many ethical questions and undermines the notion of health as a universal right.

Important elements of the right to health are available and accessible health‐care facilities and hospital care. Health‐care system priorities are commonly based on efficiency and equity in relation to the distribution of health and health care. However, there are no guarantees that, for example, a patient in need of ambulance care has access to such care, even in acute cases. There are challenges related to balancing the right to health with prioritizing health fairly.^23^ Claims on every patient's right to health care may be in contrast to political realities in most welfare states. This might be a practical concern related to the costs of health‐care provision. Thus, there may be a conflict between human rights and practical concerns of accessibility of health‐care services. Costs and available resources may limit available health‐care services, leading to some patients not benefiting from health care when needed, as described in this study's findings. Prioritizing in health care is a complex issue, particularly in light of human rights and the right to health. Yamin and Norheim^24^ state that all health‐care priority setting includes life or death choices. Internationally, health‐care systems based on general progressive income taxation tend to be the most equal,^24^ like Sweden. Considering health care as a product related to a market is inconsistent with human rights. WHO controversial discussions on universal coverage vs universal access can have contributed to detaching health from human rights by associating it with financial constraints.^16^ Still, it is a challenge to secure equal chances for all to access care when needed, as a core principle according to the right to health, causing dilemmas in health‐care priority settings. In line with Yamin and Norheim,^24^ we argue that a human rights approach is relevant to discussions and priority setting in health care.

The patient complaints analysed in this study point to a need for a more person‐centred approach towards patients in need of health care. Aasen and Dahl^12^ discuss problems related to paternalistic patterns and the imbalance in power medicine has in relation to the position of the patient. Correspondingly, Gallagher and Mazor^7^ underscore the importance of the perspective of patients and families. They point to a need for expanding our perspective beyond the technical execution of health care to include the perspective of patients. Patient‐centred care is reliant on the attitudes and approaches of health‐care professionals. Negative attitudes of health‐care professionals can have an impact on patients’ experiences and the quality of care. For instance, mental illness‐related stigma in health care and among health‐care professionals can create barriers to access and quality care.^25^ Considering health as a human right requires attention to attitudes and approaches of health‐care professionals. Individual's ability to choose, socio‐economic status and income are becoming important factors in decision making, and the value of autonomy as a basic principle is not in question. However, personal autonomy in health‐care decision making is not to be confused with dignity in human rights.^19^ More attention to the patient's perspective is required, particularly those in vulnerable situations. It should be noted that many things go well in health care, were human rights are sufficiently met and human dignity upheld. This study has however focused on challenges from patient complaints. A large‐scaled population‐based survey might establishe the frequency of violations to human rights.

### 4.1 Strengths and limitations

A major strength of the present study is the randomized sample from a large database of patient complaints. However, these formal patient complaints might only be the tip of the iceberg as there may be a large number of patients who never make formal complaints. The Swedish health‐care context might be a limitation from an international perspective, and the reader must judge if findings are transferable to their own contexts.

To uphold the rigour and validity of results, we strived to establish reflexivity and credibility during data gathering and analysis.^20^ During the analysis, we reflected on meanings and emerging themes. We questioned our understanding of the data and discussed the findings among the researchers so as not to take anything for granted. Researcher triangulation adds to the strength of the analyses and ensuing results. Original data verified themes in the results.

The complaints analysed in this study were related to different health‐care sectors. From hospital care sectors, psychiatric health‐care services stood out from the data. This study has however not focused on differences in complaints from various sectors. This might be interesting to explore in future studies.

## 5 CONCLUSION

This paper presented analyses of formal patient's complaints on health‐care services from a human rights perspective. In spite of the fact that human rights are universal, it can be concluded that these rights are being breached in relation to available and accessible health‐care services, health‐care services of good quality and dignity and equality in health care for some individual patients. Questions remain regarding how to safeguard the right to health for all. What efforts are needed to make health‐care services available and accessible? What can welfare states like Sweden afford? According to the Sustainable Development Goals, it is important to promote health and well‐being at all ages. Still, the results point to areas of improvements. Human rights to health and health care cannot be taken for granted. Patients’ rights are legislatively unclear in the Swedish context. Further debate, education and research are needed.

## ETHICAL CONSIDERATIONS

Ethical approval was obtained from the Regional Ethical Review Board in Gothenburg (DNo. 951‐15) and conforms to the Declaration of Helsinki. All data collected were made anonymous by the PAC, and no names or other personal information of patients, relatives, nurses or other persons in the complaints were obtained.

## CONFLICT OF INTEREST

No conflicts of interest have been declared.

## Data Availability

The data that support the findings of this study are available from the corresponding author upon reasonable request. The data are not publicly available due to privacy or ethical restrictions.
